# Uncoupling of sgRNAs from their associated barcodes during PCR amplification of combinatorial CRISPR screens

**DOI:** 10.1371/journal.pone.0197547

**Published:** 2018-05-25

**Authors:** Mudra Hegde, Christine Strand, Ruth E. Hanna, John G. Doench

**Affiliations:** Broad Institute of MIT and Harvard, Cambridge, Massachusetts, United States of America; University of Helsinki, FINLAND

## Abstract

Many implementations of pooled screens in mammalian cells rely on linking an element of interest to a barcode, with the latter subsequently quantitated by next generation sequencing. However, substantial uncoupling between these paired elements during lentiviral production has been reported, especially as the distance between elements increases. We detail that PCR amplification is another major source of uncoupling, and becomes more pronounced with increased amounts of DNA template molecules and PCR cycles. To lessen uncoupling in systems that use paired elements for detection, we recommend minimizing the distance between elements, using low and equal template DNA inputs for plasmid and genomic DNA during PCR, and minimizing the number of PCR cycles. We also present a vector design for conducting combinatorial CRISPR screens that enables accurate barcode-based detection with a single short sequencing read and minimal uncoupling.

## Introduction

The development and integration of oligonucleotide synthesis techniques, lentiviral vectors, and massively-parallel next-generation sequencing—the ability to write, deliver, and read DNA sequences—has enabled functional annotation of genetic elements at scale across many biological systems. Massively-parallel reporter assays (MPRA) [1–4], genome-wide screens utilizing CRISPR technology [5], and single-cell RNA sequencing studies [6–8] are just some examples of experimental approaches that have employed this general framework. Often, a barcode is linked to a sequence element of interest, and thus it is imperative to understand and minimize potential sources of false calls, that is, the uncoupling of the element from its intended barcode.

False calls in barcode-based pooled screening may arise through several distinct mechanisms. When barcodes are amplified by PCR, nucleotide misincorporation by the polymerase can lead to single nucleotide errors in barcodes; miscalls during sequencing similarly may lead to barcode changes. However, these error modes can be mitigated by ensuring that barcodes are separated by an appropriate Hamming distance [9]; barcodes altered by PCR or sequencing errors will therefore appear as unexpected sequences that can be flagged and removed prior to analysis.

It has also been previously reported that barcodes used to identify open reading frames (ORFs) can uncouple from the associated ORF during the process of lentiviral production and infection, a requisite step for most pooled screening strategies [10]. Furthermore, vectors used for single-cell RNA sequencing of CRISPR screens have recently been reported to undergo similar uncoupling between the single guide RNA (sgRNA) and its associated barcode [11–13]. Other assays that rely on barcodes are also susceptible to uncoupling. In MPRA, for example, promoter or enhancer variants are typically tagged with a transcribed barcode, which is then used to infer the identity of the variant that led to expression changes [1–4]. Similarly, screening approaches that use unique molecular identifiers (UMIs) to obtain an absolute count of cells receiving a perturbation such as an sgRNA may be susceptible to uncoupling between the UMI and the sgRNA, potentially leading to an inflated estimate of diversity [14,15]. Recently, numerous approaches to combinatorial CRISPR screens have been described, for which accurate quantitation of two unique sgRNA sequences in the same vector presents the same challenge [16–21].

## Results

We recently developed a combinatorial screening approach, dubbed “Big Papi,” which uses orthologous Cas9 enzymes from *S*. *aureus* and *S*. *pyogenes* to achieve combinatorial genetic perturbations in pooled screens [19]. Cells that already express *S*. *pyogenes* Cas9 (SpCas9) are transduced with a single Big Papi vector, which delivers *S*. *aureus* Cas9 (SaCas9) and both an SpCas9 sgRNA and an SaCas9 sgRNA. In our original implementation, the two sgRNAs were separated by ~200 nucleotides (nts), such that both could be read out with a single sequencing read, albeit a relatively long and thus more expensive sequencing run. In order to increase the cost effectiveness of the method, we set out to reduce the required read length by incorporating barcodes into the oligonucleotides used to create these pooled libraries. However, given concerns of uncoupling, we sought to examine the fidelity of our barcoding system.

**Fig 1:**
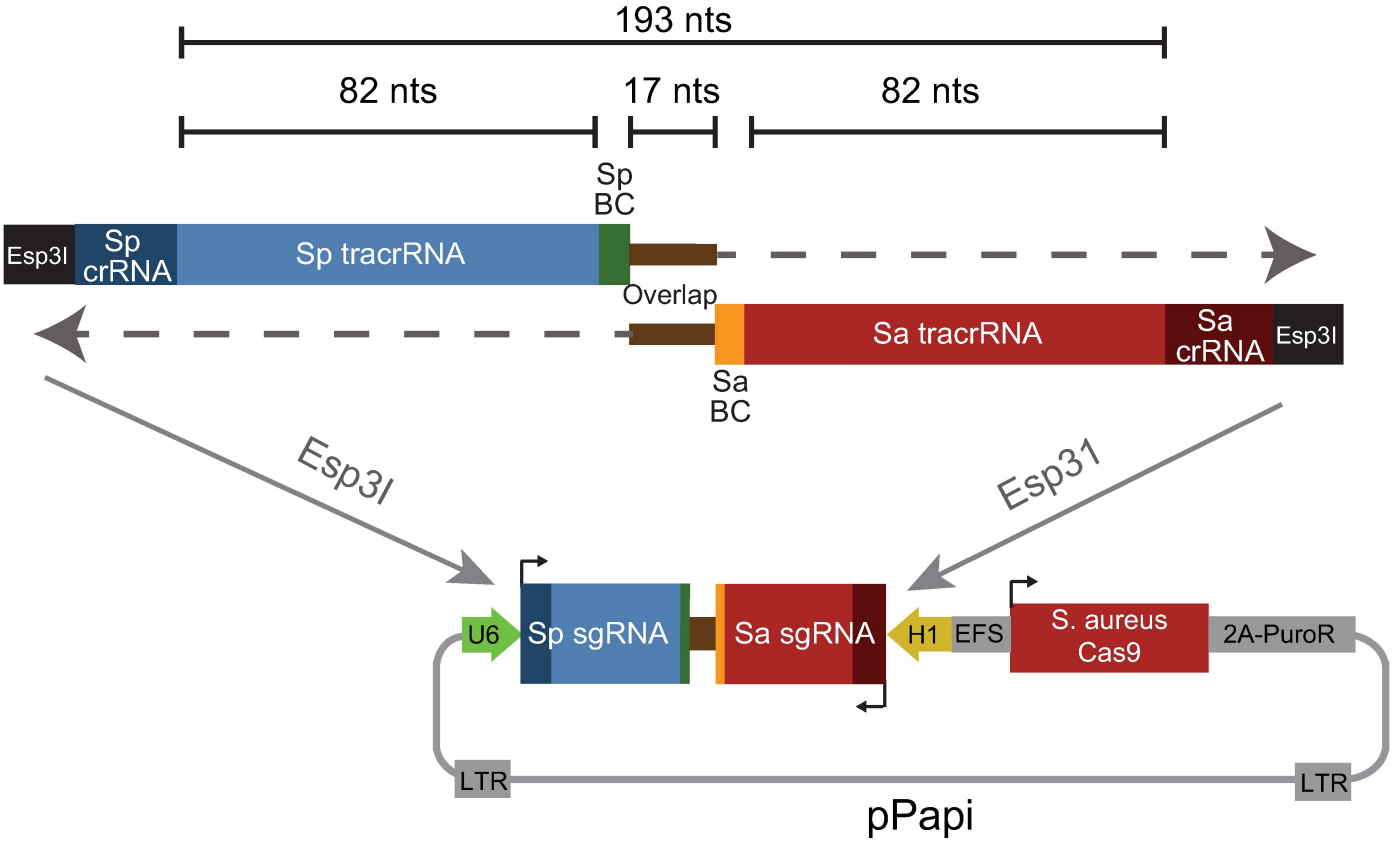
Schematic of oligonucleotide design and combinatorial library cloning strategy proposed in this study. BC: barcode.

We designed a set of hexamer barcodes with a Hamming distance of at least 2 and incorporated these barcodes into each of the sgRNA-containing oligonucleotides, immediately adjacent to the complementary regions at the 3’ end of each oligonucleotide necessary for overlap extension (Fig 1). This design places the barcodes 17 nts apart and thus requires a read length of only 29 nts to determine the combination of sgRNAs. To test the frequency of barcode uncoupling with this design, we synthesized 2 sets of 57 oligonucleotides, one for SpCas9 and one for SaCas9. To create a pooled library, we would normally mix together all the oligonucleotides to create 57 x 57 = 3,249 combinations, by performing one pooled overlap extension reaction. Here, however, only oligonucleotides from analogous wells were mixed together—e.g. well A1 oligonucleotides for SpCas9 and SaCas9 were mixed together, etc.–for a total of 57 combinations, and 57 individual overlap extension reactions were performed in parallel. The resulting dsDNA products were pooled and cloned into the pPapi vector by Golden Gate cloning (see Methods). This library is thus sensitive both to uncoupling of barcodes from their associated sgRNAs, as well as to unintended combinations of sgRNAs or barcodes, as only a small fraction (57 ÷ 3,249 = 1.7%) of all potential SaCas9/SpCas9 sgRNA combinations should be present.

From the plasmid DNA (pDNA) library, we generated lentivirus and infected it into A375 cells expressing SpCas9. One week after infection, sufficient time to allow any residual pDNA carried over from the production of lentivirus to degrade and dilute [10], we prepared genomic DNA (gDNA). We then performed PCR as previously described for standard pooled screens [22], using 28 cycles for both the pDNA (10 ng input) and gDNA (10 μg input) and primers that amplified both of the sgRNAs and their associated barcodes. We sequenced the resulting products with a single end read of sufficient length (300 nts) to capture all relevant sequences.

**Fig 2:**
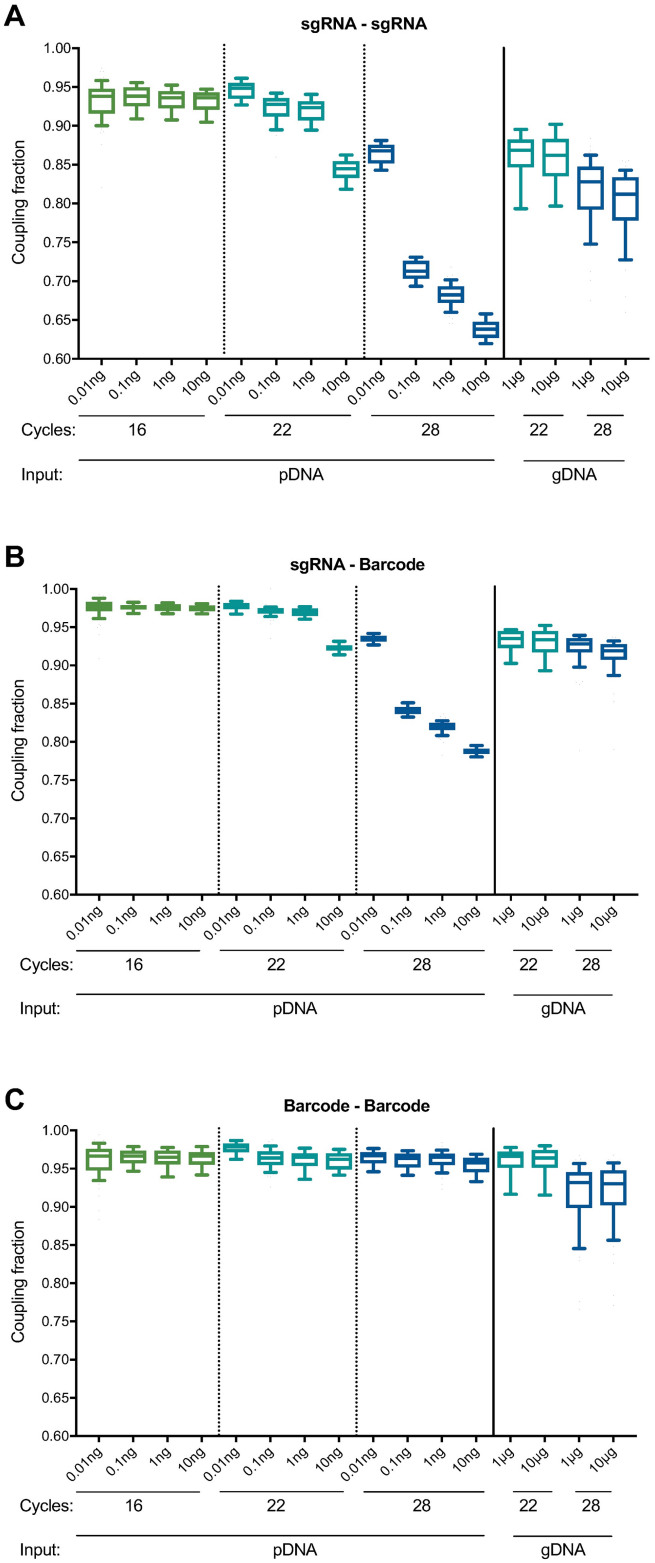
Comparison of uncoupling across sample types, input amounts, and number of PCR cycles. (A) Uncoupling between SaCas9 sgRNAs and SpCas9 sgRNAs under various PCR conditions. Each box represents 57 paired sgRNAs, plotting the fraction of reads for which the sgRNAs were correctly paired. The line represents the median, the box the 25th and 75th percentiles, and the whiskers the 10th and 90th percentiles. Our initial PCR conditions (28 cycles with 10 ng pDNA and 10 μg gDNA) led to substantial uncoupling. (B) Uncoupling between sgRNAs and their associated barcodes under various PCR conditions. (C) Uncoupling between barcodes under various PCR conditions. Box and whisker plots in (B) and (C) are the same as in (A).

We analyzed the sequencing reads for evidence of uncoupling between sgRNAs (e.g. an SpCas9 sgRNA from well A1 appearing in combination with an SaCas9 sgRNA from any other well). We found substantially more uncoupling in the pDNA sample than in the gDNA sample, with only 64% of sgRNAs appearing with their correctly-matched sgRNA for the pDNA sample (10 ng, 28 cycles), whereas 81% were correctly paired in the gDNA sample (10 μg, 28 cycles; Fig 2A and S1 Table). Likewise, we examined uncoupling between sgRNAs and their associated barcodes and observed that, across the 57 sgRNAs for each Cas9, a median of 79% and 92% of sgRNAs were appropriately coupled to their barcodes in the pDNA and gDNA samples, respectively (Fig 2B and S2 Table). We observed minimal barcode-barcode uncoupling with either pDNA (96% coupled) or gDNA (93% coupled) (Fig 2C and S3 Table), which are separated by only 17 nts. These results, whereby the pDNA generally showed more extensive uncoupling than the gDNA, were unexpected, as only the gDNA sample had been packaged into lentivirus and integrated into cells, steps previously suggested to generate uncoupling [10–13,23]. Moreover, the same pDNA had been used to generate the lentivirus infected into cells, suggesting that the pDNA uncoupling had not occurred prior to lentiviral production.

We noted that one potentially relevant difference between the two samples was the number of template molecules: 10 ng of pDNA contains ~500-fold more template molecules than 10 μg of gDNA (8.1x10^8^ vs. 1.5x10^6^ template molecules, respectively; see Methods for calculations). We also considered that the number of PCR cycles could affect uncoupling. Thus, we asked whether starting with comparable numbers of template molecules or varying the number of PCR cycles could alter the observed rates of uncoupling.

In both pDNA and gDNA samples, we found that decreasing both the number of cycles and template molecules decreased uncoupling. When using 22 cycles of PCR and approximately equal numbers of template molecules (10 pg pDNA, 10 μg gDNA), we observed that 95% and 86% of sgRNAs were correctly coupled, respectively (Fig 2A and S1 Table). Likewise, under these PCR conditions, a median of 98% of reads showed appropriate coupling of sgRNAs and their associated barcodes in the pDNA sample, whereas the gDNA showed 93% correct coupling (Fig 2B and S2 Table). Barcode-barcode uncoupling was again minimal, with 98% and 96% correct coupling for pDNA and gDNA, respectively (Fig 2C and S3 Table). Thus, when the amounts of template were normalized, the results were consistent with some uncoupling occurring during lentiviral production. We also observed less uncoupling with 22 cycles of PCR compared to 28 cycles.

**Fig 3:**
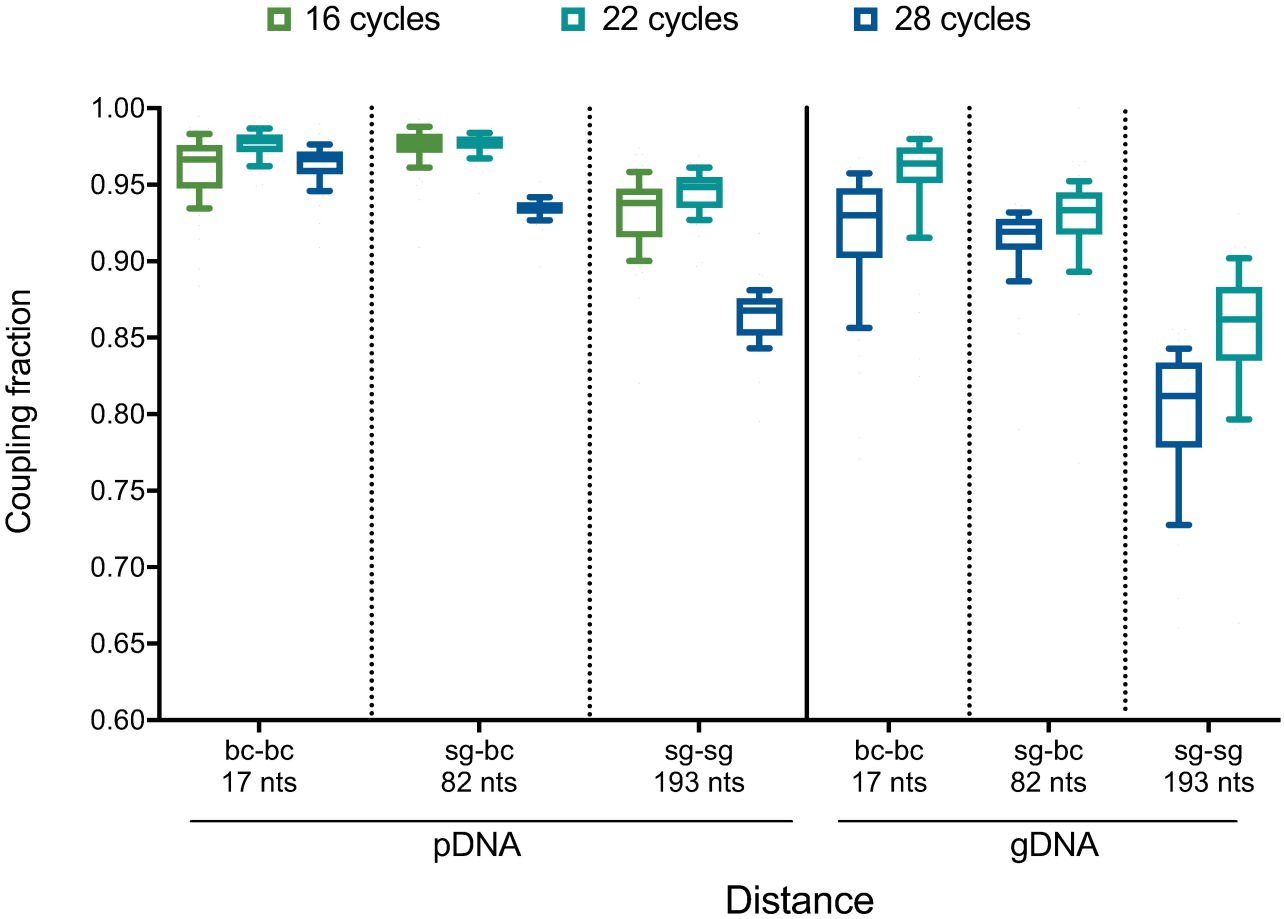
Comparison of uncoupling at various distances between linked elements. Data are replotted from Fig 2. Uncoupling between barcodes (separated by 17 nts), an sgRNA and its associated barcode (82 nts), and sgRNAs (193 nts). PCR was performed at either 16, 22, or 28 cycles using 10 pg pDNA (left) or 10 μg gDNA (right).

These results implicate the PCR step as a large source of uncoupling under conditions of either higher template amounts or cycling number. One potential mechanism to explain these observations is abortive products, in which the polymerase falls off the template after it has amplified one sgRNA (or barcode) but has not finished the product. In this scenario, which has been previously observed [24,25], the 3’ end of this abortive product is capable of serving as a primer in the next cycle by binding to common, intervening sequence and extending, thus coupling the initial sgRNA (or barcode) to a different, unintended sgRNA (or barcode). Such abortive products may become more common as nucleotides become more limiting, as would be the case in later cycles of PCR or with more templates of input, as more products have been formed and thus fewer free nucleotides are available. Uncoupling may also occur when the polymerase jumps between templates mid-extension [26,27]. Both mechanisms are consistent with the observation that substantially more uncoupling occurred between two sgRNAs (separated by 193 nts) than between two barcodes (separated by 17 nts) (Fig 3), as a greater intervening distance between two elements of interest increases the probability of the polymerase aborting between them.

**Fig 4:**
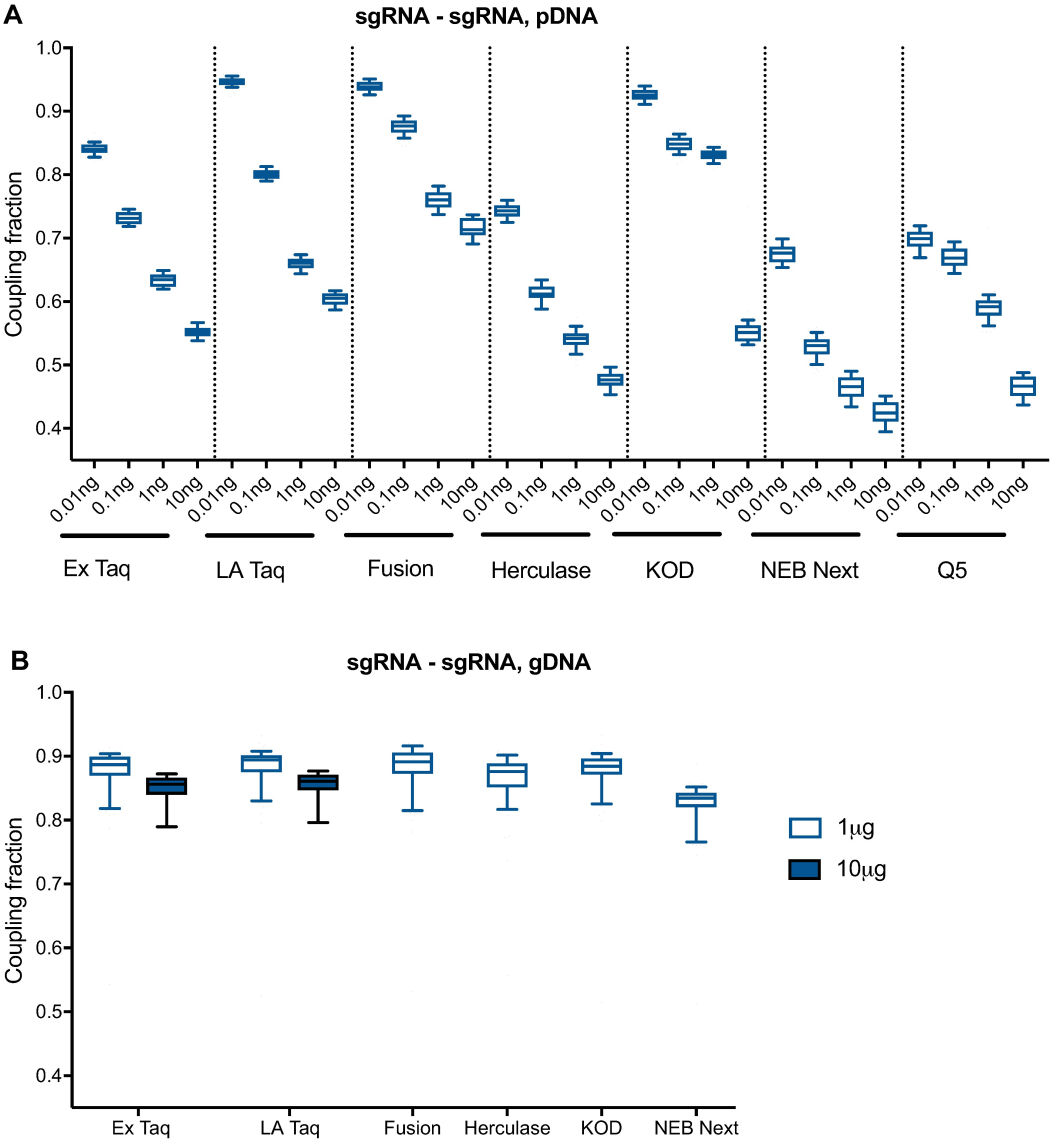
Comparison of PCR polymerases with pDNA and gDNA. (A) Uncoupling between sgRNAs, using 28 cycles of PCR and a range of pDNA inputs. (B) Uncoupling between sgRNAs, using 28 cycles of PCR and either 1 or 10 μg of gDNA. Only Ex Taq and LA Taq produced a product with 10 μg of gDNA input.

To test whether the PCR polymerase had an effect on uncoupling, we compared 7 polymerases, including the previously-used Ex Taq. We first tested each polymerase on pDNA with 28 cycles of PCR, which was the most sensitive condition to uncoupling. Using a range of template inputs, we found that Fusion, KOD, and LA Taq had the highest performance, with a median sgRNA-sgRNA coupling fraction of >90% with 10 pg of pDNA input (Fig 4A and S4 Table). Ex Taq also performed fairly well, with 84% correctly coupled sgRNAs under these same conditions; Herculase, NEB Next, and Q5 showed comparatively poor performance, with <80% correct coupling at all pDNA inputs. We observed a similar trend of polymerase performance with sgRNA-barcode and barcode-barcode coupling. Although we cannot rule out that different reaction parameters would alter the relative performance of these polymerases, the results provide guidance for which polymerases may be the best first choice for applications in which uncoupling is a concern.

**Fig 5:**
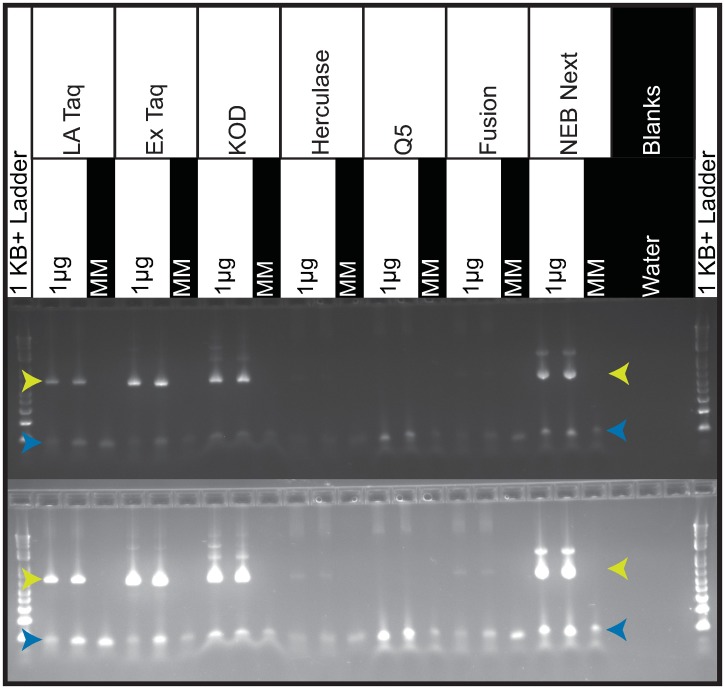
Gel image of PCR products comparing polymerase performance with 1 μg of gDNA input. Product bands are 650nt and indicated with yellow arrows. Blue arrows indicate unused primer bands. Both images are of the same 2% Agarose gel at two different exposures. In both exposures, LA Taq, Ex Taq, KOD and NEB Next produce robust product bands. Herculase and Fusion produce very faint product bands only visible with overexposure. Q5 produced no visible band even with overexposure. The ladder is the 1KB Plus (ThermoFisher, 10787018).

Subsequently, we tested each polymerase on gDNA, again using 28 cycles of PCR to sensitively detect uncoupling. With 1 μg of gDNA input, LA Taq, Ex Taq, KOD and NEB Next gave the best amplification, whereas other polymerases produced less product (Fig 5). However, only Ex Taq and LA Taq successfully amplified from 10 μg of gDNA, as expected based on the recommended amplification conditions; most polymerases perform better with less DNA template. With 1 μg of gDNA input, LA Taq, Fusion, Ex Taq, KOD and Herculase performed similarly, with a median sgRNA-sgRNA coupling fraction of ~89%. With 10 μg of gDNA input, both Ex Taq and LA Taq had a median sgRNA-sgRNA coupling fraction of 86% (Fig 4B and S4 Table), and a barcode-barcode coupling fraction of 99%. Given that combinatorial screens require a large number of cells and thus result in large amounts of gDNA, Ex Taq, which tolerates higher amounts of gDNA in a reaction and shows little uncoupling under these conditions, especially between barcodes, remains our preferred polymerase.

## Discussion

**Fig 6:**
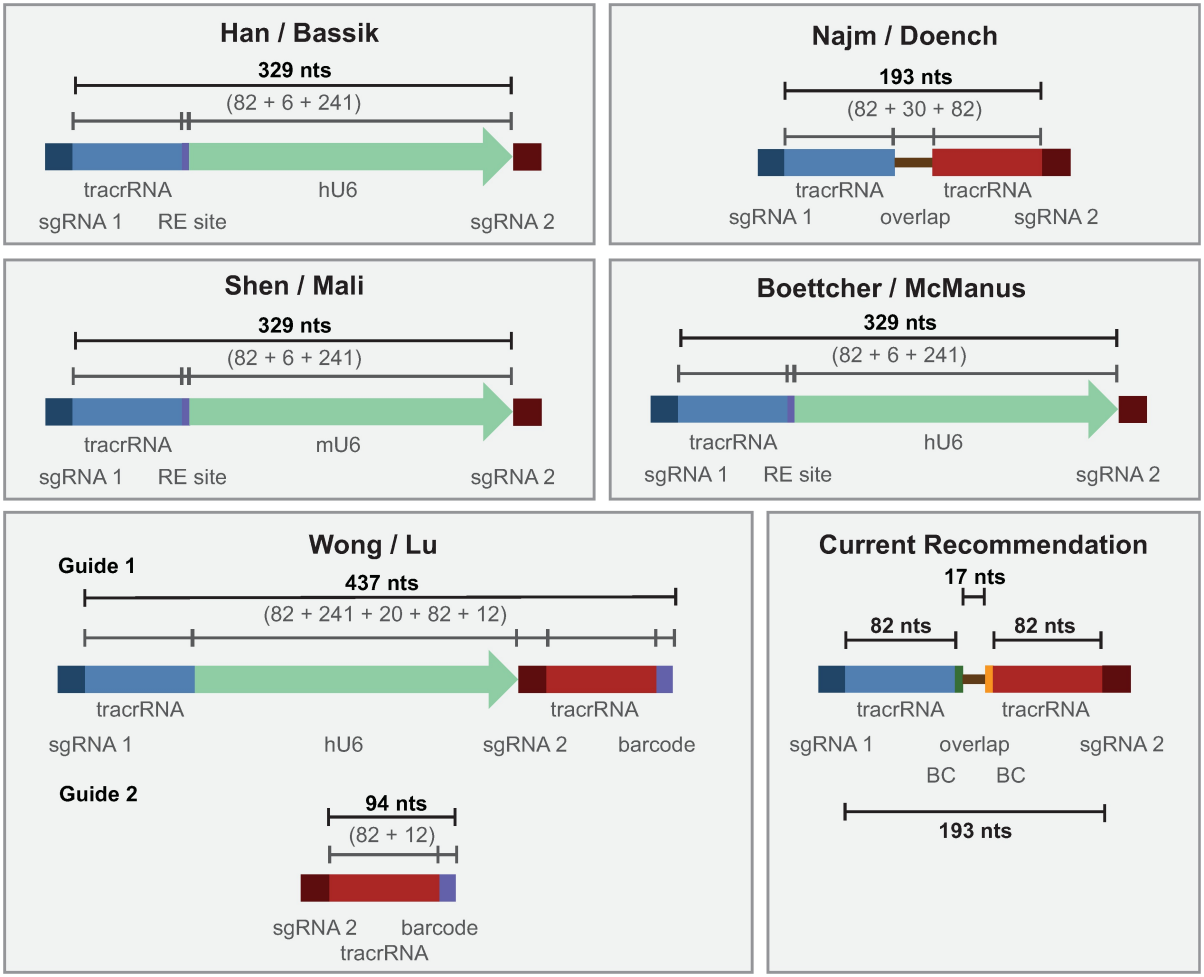
Schematics of various vector designs used for combinatorial CRISPR screens. See also Table 1. BC: barcode. RE: restriction enzyme.

The importance of PCR cycle number and template DNA input for PCR-based recombination has been previously observed [24,25,28–33], but is of particular relevance given the current interest in barcode-based pooled screening. Multiple designs have been used to express pairs of sgRNAs used in combinatorial CRISPR screens (Fig 6, Table 1), and all require performing PCR to retrieve the sgRNAs or barcodes from the genomic DNA. Our results suggest several optimizations to minimize PCR-based uncoupling. First, the distance between linked elements should be kept to a minimum; in current approaches, the distance between relevant elements has varied widely. Alternative experimental designs can also be used to make shuffling easily detectable; for example, if only specific sgRNA pairs are programmed into a library, rather than all possible combinations, any unexpected chimeric reads can be easily filtered out [21]. Second, when amplifying pDNA to serve as a measure of initial library abundance, it is important to use a similar amount of template molecules as present in the gDNA samples; for 10 μg of gDNA from a human cell, this corresponds to approximately 20 pg of pDNA. Finally, our results demonstrate that uncoupling increases with the number of PCR cycles; cycle number should therefore be kept to the minimum required to produce a sufficient product. When a large number of cycles are required, a nested or reconditioning PCR approach may reduce shuffling by replenishing dNTPs and primers, which presumably reduces the likelihood of abortive products [33]. Previous reports have also recommended lengthening the elongation step [24,25]. Regardless, shuffling rates should be determined empirically for any new vector system by a corresponding arrayed experiment.

**Table 1:** PCR conditions from combinatorial sgRNA studies.

Study	Distance between variable elements	PCR cycles
Najm / Doench [19]	sgRNA-1 to sgRNA-2: 194 nts	Single PCR: 28 cycles
Han / Bassik [16]	sgRNA-1 to sgRNA-2: 329 nts*	Nested PCR: 1) 18 cycles 2) 24 cycles 42 cycles total
Shen / Mali [17]	sgRNA-1 to sgRNA-2: 329 nts*	Nested PCR: 1) 21–26 cycles 2) 7–8 cycles 28–34 cycles total
Wong / Lu [18]	sgRNA-1 to barcode-1: 437 nts* sgRNA-2 to barcode-2: 94 nts*	Nested PCR: Cycle numbers not provided
Boettcher / McManus [20]	sgRNA-1 to sgRNA-2: 329 nts*	Nested PCR: 1) 16 cycles 2) 16 cycles 32 cycles total
Current design (this study)	sgRNA-1 to barcode-1: 82 nts sgRNA-2 to barcode-2: 82 nts barcode-1 to barcode-2: 17 nts	Single PCR: 22 cycles

* Distances between sgRNAs or between sgRNAs and their associated barcodes calculated from other studies are estimates based on the provided vector schematics. Some additional non-annotated sequences may be present in some designs.

These results also reinforce previous findings that recombination during lentiviral replication, a distance dependent factor, is another important source of uncoupling [10–13,23]. Thus, minimizing the distance between elements, which reduces the likelihood of uncoupling during both lentiviral replication and PCR, should be an important design parameter. Another recently proposed strategy to reduce recombination during lentiviral packaging is to dilute the library with a carrier plasmid during lentiviral production [12], although this approach reduces viral titer by about 100-fold and thus is likely not practical for many cell-based applications.

Our current preferred combinatorial vector design has a short distance between the sgRNA and its barcode, 82 nts (the length of the tracrRNA), which results in minimal uncoupling during lentiviral production. Further, the two barcodes are only 17 nts apart, and thus there is little chance for uncoupling between barcodes during PCR retrieval of the cassette following a screen. This design should help to minimize this source of noise in combinatorial genetic screens. Additionally, these results provide guidance for optimizing many other experimental settings that use a barcode to track a sequence element of interest.

## Methods

### Vectors

The pPapi plasmid used for dual expression of sgRNAs was previously described [19] and is available from Addgene (#96921).

### Library production

Two sets of oligonucleotides were ordered from Integrated DNA Technologies (IDT, Iowa). One set generates SpCas9 sgRNAs that will be expressed from the U6 promoter in pPapi, the other set generates SaCas9 sgRNAs that will be expressed from the H1 promoter. Each oligonucleotide is 139 nts in length and were ordered as Ultramers, delivered at a final concentration of 5 μM. Oligonucleotides were mixed by well—e.g. SpCas9 A1 mixed with SaCas9 A1, SpCas9 A2 mixed with SaCas9 A2, etc.–using 2 μL of each oligonucleotide; 6 μL water; 10 μL NEB Next 2x master mix (New England Biolabs M0541L). The 57 reactions were overlap-extended as follows:

1. 98°C for 3 minutes
2. 98°C for 30 seconds; 48°C for 30 seconds; 72°C for 1 minute, for 12 cycles
3. 72°C for 5 minutes

The 57 reactions were then purified by adding 5 μL of each reaction to 1.5 mL buffer PB and proceeding with a PCR spin column purification (Qiagen 28104).

To generate pooled libraries in which combinations are not separated by individual wells, we recommend the following:

1. Pool all SpCas9 oligonucleotides at 5 μM; pool all SaCas9 oligonucleotides at 5 μM.
2. To 10 μL 10x Ex Taq buffer and 70 uL water, add 5 μL SpCas9 pool and 5 μL SaCas9 pool.
3. Pre-warm heat block to 95°C, add mixture, turn off heat block, and allow to slowly cool to room temperature (~2 hours). When done, turn heat block back on as a token of good lab citizenship, although this will increase the experiment’s carbon footprint.
4. Add 8 μL dNTPs, 2 μL Ex Taq (Takara RR001A), onto thermocycler: 48° for 40 minutes, 72° for 20 minutes.
5. Purify by adding to 500 μL buffer PB and proceeding with a PCR spin column purification.

The resulting dsDNA is then ligated into the BsmBI-digested pPapi vector using Golden Gate assembly:

5 μL Tango Buffer (ThermoFisher)

5 μL DTT (stored at -80°C and used once, 10 mM stock)

5 μL ATP (stored at -80°C and used once, 10 mM stock)

500 ng pPapi vector, pre-digested with Esp3I or BsmBI, gel-extracted, and isopropanol-precipitation purified

100 ng dual sgRNA dsDNA insert

1 μL Esp3I (ThermoFisher ER0452)

1 μL T7 ligase (Enzymatics, 3,000 Units / μL L6020L)

Up to 50 μL water

Cycle 100x (overnight): 5 minutes at 37°C, 5 minutes at 20°C.

Purify Golden Gate product by isopropanol precipitation. Per 50 μL reaction, add in order:

1 μL GlycoBlue (Ambion AM9515)
4 μL NaCl, 5M
55 μL isopropanol

1. Vortex, and incubate at room temperature for 15 minutes.
2. Centrifuge at >10,000g for 15 minutes at room temperature.
3. Remove liquid, avoiding the pellet (it is okay to leave a little liquid behind).
4. Add 950 μL 70% EtOH, vortex, centrifuge for 5 minutes at room temperature, remove liquid.
5. Repeat step (c).
6. Centrifuge for 1 minute and remove any residual liquid with a fine-tipped pipette (e.g. P200 or smaller); allow to air dry for 1 minute.
7. Resuspend with 10 μL water or TE, on ice. Flick the tube and briefly centrifuge as needed.

To transform the library into *E*. *coli*, we recommend STBL4 cells (Invitrogen 11635018). Add 10 μL of isopropanol-precipitated DNA to 100 μL electrocompetent cells. This step will need to be scaled as library size increases.

### Virus production

Pooled library virus was made using the same large scale T175 flask method used previously [19]. Briefly, 24 hours pre-transfection, 18 × 10^6^ HEK293T cells were seeded into a 175 cm^2^ tissue culture flask with 24 mL of DMEM + 10% FBS. Next day, one solution of Opti-MEM (Corning, 6 mL) and LT1 (Mirus, 305 μL) was combined with a DNA mixture of the packaging plasmid pCMV-VSVG (Addgene 8454, 5 μg), psPAX2 (Addgene 12260, 50 μg), and sgRNA-containing vector (pPapi, 40 μg). This mixture was incubated for 20–30 min at room temperature, during which media was changed on the HEK293Ts. Following incubation, the transfection mixture was added dropwise to cells. The cells were incubated for 6–8 h, after which time media was replaced with DMEM + 10% FBS, supplemented with 1% BSA. 36 hours post-media replacement, virus was harvested.

### Cell culture

A375 cells were obtained from the Cancer Cell Line Encyclopedia. Cells were cultured in RPMI + 10% FBS, routinely tested for mycoplasma contamination and maintained in a 37 °C humidity-controlled incubator with 5.0% CO_2_. Cells were maintained in exponential phase growth by passaging every 2 or 3 days. Cell lines were maintained without antibiotics, and supplemented with 1% penicillin/streptomycin post-lentiviral infection. The A375 Cas9 derivative was made by transducing with the lentiviral vector pLX_311-Cas9, which expresses blasticidin resistance from the SV40 promoter and Cas9 from the EF1α promoter (Addgene 96924).

### Infection optimization

A375 cells stably expressing SpCas9 were infected as described previously [19].

### Genomic DNA preparation

Genomic DNA (gDNA) was isolated using the QIAamp DNA Blood Maxi Kit (Qiagen) as per the manufacturer’s instructions. Resulting gDNA was quantitated by UV Spectroscopy (Nanodrop). Going forward, we recommend the use of Nucleospin Blood XL kits (Macherey-Nagel, 740950) for gDNA isolation, and the use of Qubit with the dsDNA BR kit (Invitrogen Q32850) to quantitate concentration.

### Calculations for templates of input

gDNA:

$$1\;template=1\;cell\;(assuming\;3\times 10^{9}\;basepairs\;per\;cell)=6.6\;pg\;gDNA$$

$$10\mu g\;gDNA\times \frac{1\;cell}{6.6\;pg\;gDNA}=1.5\times 10^{6\;}template\;molecules$$

pDNA:

$$1\;template=1\;plasmid\;of\;12.3kB\;(7.5\times 10^{6}\frac{g}{mol})=1.24\;pg\;pDNA$$

$$10\mu g\;gDNA\times \frac{1\;plasmid}{1.24\;pg\;pDNA}=8.1\times 10^{5}template\;molecules$$

### PCR and sequencing methods

Dual sgRNA cassettes were PCR-amplified and barcoded with sequencing adaptors using Ex Taq polymerase except where otherwise specified. When we tested alternative polymerases, we also used LA Taq HS, KOD HS, Herculase HS, Q5 and PfuUltra Fusion polymerase kits following manufacturer recommendations for PCR amplification conditions (Table 2). For kits that did not provide dNTPs, the suggested concentration of dNTPs was added using the 2.5 mM per dNTP stock provided in Takara’s Ex Taq kit.

**Table 2:** PCR conditions for various polymerases.

Polymerase Kit	Manufacturer and Catalog Number	Master Mix	Polymerase	Buffer	Mg^2+^	dNTP	P5	P7	Water + DNA (μL)	Total Volume (μL)
Ex Taq	Takara RR001A		1.5μL at 5U/μL	10μL of Ex Taq Mg2+ 10X buffer		8μL at 2.5mM each of from Ex Taq Kit	0.5 μL at 100μM	10 μL at 5μM	70μL	100μL
						(0.2mM)	(0.5μM)	(0.5μM)		
LA Taq HS	Takara RR042A		1μL at 5U/μL	10μL of LA Taq HS Mg2+ 10X buffer		16μL at 2.5mM each from LA Taq HS kit	5 μL at 100μM	10 μL at 5μM	58 μL	100μL
						(0.4mM)	(0.5μM)	(0.5μM)		
KOD HS	Novagen 71086–5		2μL at 1U/μL	10μL of KOD HS 10X buffer	6μL of 25mM MgSO_4_	10μL at 2mM each from KOD HS kit	0.3μL at 100μM	6μL at 5μM	65.7 μL	100μL
						(0.2mM)	(0.3μM)	(0.3μM)		
Herculase HS	Agilent Technologies Inc 600310		1μL at 5U/μL	10μL of Herculase HS 10X buffer		8μL at 2.5mM each from Ex Taq Kit	0.25μL at 100μM	5μL at 5μM	75.75 μL	100μL
						(0.2mM)	(0.25μM)	(0.25μM)		
Q5	New England Biolab M0493L		1μL at 2U/μL	20μL of 5X Q5 Reaction Buffer		8μL at 2.5mM each from Ex Taq Kit	0.5 μL at 100μM	10 μL at 5μM	60.5 μL	100μL
						(0.2mM)	(0.5μM)	(0.5μM)		
PfuUltra Fusion	Agilent Technologies Inc 600670		2μL	10μL of PfuUltra fusion HS 10X buffer		10μL at 2.5mM each of Ex Taq Kit	0.2μL at 100μM	4μL at 5μM	73.8μL	100μL
						(0.25mM)	(0.2μM)	(0.2μM)		
NEB Next	New England Biolabs M0541L	50μL of 2X Master Mix					0.5 μL at 100μM	10 μL at 5μM	39.5μL	100μL
							(0.5μM)	(0.5μM)		

All volumes are calculated for one 100 μL volume reaction.

P5/P7 primers were synthesized at Integrated DNA Technologies (IDT):

*Forward (P5)* 5’**AATGATACGGCGACCACCGAGATCT**ACACTCTTTCCCTACACGACGCTCTTCCGA TCT**[s]**TTGTGGAAAGGACGAAAC*A*C*C*G

*Reverse (P7)* 5’**CAAGCAGAAGACGGCATACGAGA**T*NNNNNNNN*GTGACTGGAGTTCAGACGTGT GCTCTTCCGATCTCCAATTCCCACTCCTTTCAA*G*A*C*C

**P5/P7 flow-cell attachment sequence**

Illumina sequencing primer

**[Stagger region]**

*Barcode* *region*

Vector primer binding sequence

* between bases indicate phosphorothioate linkages

PCR cycling conditions:

1. 1 minute at 95°C
2. 30 seconds at 94°C, 30 seconds at 52.5°C, 30 seconds at 72 °C, for *n* cycles
3. 10 minutes extension at 72 °C.

Following PCR, samples were purified with Agencourt AMPure XP SPRI beads (Beckman Coulter A63880) according to manufacturer’s instructions. In cases where gel images following PCR suggested a wide range of DNA yield per well, wells with similar band strengths were purified together in sub-pools. Each purified sub-pool was quantitated with UV spectroscopy (Nanodrop) and pooled into a master sequencing pool such that each PCR well contributed approximately equally to the final master pool. The master pools were sequenced on a MiSeq sequencer (Illumina) with 300 nt single-end reads, loaded with a 5% spike-in of PhiX DNA.

### Analysis

Reads of the first sgRNA were counted by searching for CACCG, part of the vector sequence that immediately precedes the 20-nucleotide U6 promoter-driven SpCas9 sgRNA. The sgRNA sequence following this search string was mapped to a reference file with all SpCas9 sgRNAs in the library. Reads of the SpCas9 sgRNA-associated six-nucleotide barcodes were then counted by searching for part of the SpCas9 tracr sequence that precedes the barcode. The barcode was then mapped to a reference file with all SpCas9 sgRNA-associated barcodes.

Reads for the H1 promoter-driven SaCas9 sgRNA were counted by searching for part of the reverse complement of the SaCas9 tracr sequence (CTTAAAC). The 21-nucleotide sgRNA sequence following the search string was mapped to the reference file with all SaCas9 sgRNAs in the library. Reads for the six-nucleotide barcode associated with the SaCas9 sgRNA were then counted by searching for part of the overlap extension region preceding the barcode. The barcode was then mapped to the reference file with all SaCas9-associated barcodes.

The coupling fractions can be calculated using the python script found in this Github link: https://github.com/mhegde/coupling-fraction-calculation.

## Supporting information

S1 Table Raw read counts for sgRNA-sgRNA coupling at various cycle numbers and DNA input. (XLSX)

S2 Table Raw read counts for sgRNA-barcode coupling at various cycle numbers and DNA input. (XLSX)

S3 Table Raw read counts for barcode-barcode coupling at various cycle numbers and DNA input. (XLSX)

S4 Table Raw read counts for sgRNA-sgRNA coupling with various polymerases and DNA input. (XLSX)

## References

[1] Maricque BB, Dougherty JD, Cohen BA. A genome-integrated massively parallel reporter assay reveals DNA sequence determinants of cis-regulatory activity in neural cells. Nucleic Acids Res. 2017;45: e16 doi: 10.1093/nar/gkw942 28204611 10.1093/nar/gkw942 PMC5389540

[2] O’Connell DJ, Kolde R, Sooknah M, Graham DB, Sundberg TB, Latorre IJ, et al Simultaneous Pathway Activity Inference and Gene Expression Analysis Using RNA Sequencing. Cell Syst. Elsevier Inc.; 2016;2: 323–334. 10.1016/j.cels.2016.04.011 PMC5032147 27211859

[3] Patwardhan RP, Lee C, Litvin O, Young DL, Pe’er D, Shendure J. High-resolution analysis of DNA regulatory elements by synthetic saturation mutagenesis. Nat Biotechnol. 2009;27: 1173–1175. doi: 10.1038/nbt.1589 19915551 10.1038/nbt.1589 PMC2849652

[4] Melnikov A, Zhang X, Rogov P, Wang L, Mikkelsen TS. Massively parallel reporter assays in cultured mammalian cells. J Vis Exp. 2014; doi: 10.3791/51719 25177895 10.3791/51719 PMC4364389

[5] Doench JG. Am I ready for CRISPR? A user’s guide to genetic screens. Nat Rev Genet. 2018;19: 67–80. doi: 10.1038/nrg.2017.97 29199283 10.1038/nrg.2017.97

[6] Dixit A, Parnas O, Li B, Chen J, Fulco CP, Jerby-Arnon L, et al Perturb-Seq: Dissecting Molecular Circuits with Scalable Single-Cell RNA Profiling of Pooled Genetic Screens. Cell. Elsevier Inc.; 2016;167: 1853–1866.e17. 10.1016/j.cell.2016.11.038 PMC5181115 27984732

[7] Adamson B, Norman TM, Jost M, Cho MY, Nuñez JK, Chen Y, et al A Multiplexed Single-Cell CRISPR Screening Platform Enables Systematic Dissection of the Unfolded Protein Response. Cell. Elsevier Inc.; 2016;167: 1867–1882.e21. 10.1016/j.cell.2016.11.048 PMC5315571 27984733

[8] Datlinger P, Rendeiro AF, Schmidl C, Krausgruber T, Traxler P, Klughammer J, et al Pooled CRISPR screening with single-cell transcriptome readout. Nat Methods. 2017;14: 297–301. doi: 10.1038/nmeth.4177 28099430 10.1038/nmeth.4177 PMC5334791

[9] Hamming RW. Error Detecting and Error Correcting Codes. Bell System Technical Journal. Wiley Online Library; 1950;29: 147–160.

[10] Sack LM, Davoli T, Xu Q, Li MZ, Elledge SJ. Sources of Error in Mammalian Genetic Screens. G3. 2016;6: 2781–2790. doi: 10.1534/g3.116.030973 27402361 10.1534/g3.116.030973 PMC5015935

[11] Xie S, Cooley A, Armendariz D, Zhou P, Hon G. Frequent sgRNA-barcode Recombination in Single-cell Perturbation Assays [Internet]. bioRxiv. 2018 p. 255638 doi: 10.1101/255638 10.1371/journal.pone.0198635 PMC5991360 29874289

[12] Feldman D, Singh A, Garrity AJ, Blainey PC. Lentiviral co-packaging mitigates the effects of intermolecular recombination and multiple integrations in pooled genetic screens [Internet]. bioRxiv. 2018 p. 262121 doi: 10.1101/262121

[13] Hill AJ, McFaline-Figueroa JL, Starita LM, Gasperini MJ, Matreyek KA, Packer J, et al On the design of CRISPR-based single-cell molecular screens. Nat Methods. 2018; doi: 10.1038/nmeth.4604 29457792 10.1038/nmeth.4604 PMC5882576

[14] Michlits G, Hubmann M, Wu S-H, Vainorius G, Budusan E, Zhuk S, et al CRISPR-UMI: single-cell lineage tracing of pooled CRISPR-Cas9 screens. Nat Methods. Nature Research; 2017; doi: 10.1038/nmeth.4466 29039415 10.1038/nmeth.4466

[15] Schmierer B, Botla SK, Zhang J, Turunen M, Kivioja T, Taipale J. CRISPR/Cas9 screening using unique molecular identifiers. Mol Syst Biol. 2017;13: 945 doi: 10.15252/msb.20177834 28993443 10.15252/msb.20177834 PMC5658704

[16] Han K, Jeng EE, Hess GT, Morgens DW, Li A, Bassik MC. Synergistic drug combinations for cancer identified in a CRISPR screen for pairwise genetic interactions. Nat Biotechnol. Nature Publishing Group; 2017;35: 463–474. 10.1038/nbt.3834 PMC5557292 28319085

[17] Shen JP, Zhao D, Sasik R, Luebeck J, Birmingham A, Bojorquez-Gomez A, et al Combinatorial CRISPR–Cas9 screens for de novo mapping of genetic interactions. Nat Methods. Nature Publishing Group, a division of Macmillan Publishers Limited. All Rights Reserved.; 2017;14: 573. 10.1038/nmeth.4225 PMC5449203 28319113

[18] Wong ASL, Choi GCG, Cui CH, Pregernig G, Milani P, Adam M, et al Multiplexed barcoded CRISPR-Cas9 screening enabled by CombiGEM. Proc Natl Acad Sci U S A. National Acad Sciences; 2016;113: 2544–2549. 10.1073/pnas.1517883113 PMC4780610 26864203

[19] Najm FJ, Strand C, Donovan KF, Hegde M, Sanson KR, Vaimberg EW, et al Orthologous CRISPR-Cas9 enzymes for combinatorial genetic screens. Nat Biotechnol. 2017; doi: 10.1038/nbt.4048 29251726 10.1038/nbt.4048 PMC5800952

[20] Boettcher M, Tian R, Blau JA, Markegard E, Wagner RT, Wu D, et al Dual gene activation and knockout screen reveals directional dependencies in genetic networks. Nat Biotechnol. 2018;36: 170–178. doi: 10.1038/nbt.4062 29334369 10.1038/nbt.4062 PMC6072461

[21] Gasperini M, Findlay GM, McKenna A, Milbank JH, Lee C, Zhang MD, et al CRISPR/Cas9-Mediated Scanning for Regulatory Elements Required for HPRT1 Expression via Thousands of Large, Programmed Genomic Deletions. Am J Hum Genet. 2017;101: 192–205. doi: 10.1016/j.ajhg.2017.06.010 28712454 10.1016/j.ajhg.2017.06.010 PMC5544381

[22] Doench JG, Fusi N, Sullender M, Hegde M, Vaimberg EW, Donovan KF, et al Optimized sgRNA design to maximize activity and minimize off-target effects of CRISPR-Cas9. Nat Biotechnol. 2016;34: 184–191. doi: 10.1038/nbt.3437 26780180 10.1038/nbt.3437 PMC4744125

[23] Hu WS, Temin HM. Genetic consequences of packaging two RNA genomes in one retroviral particle: pseudodiploidy and high rate of genetic recombination. Proc Natl Acad Sci U S A. 1990;87: 1556–1560. 2304918 10.1073/pnas.87.4.1556 PMC53514

[24] Meyerhans A, Vartanian JP, Wain-Hobson S. DNA recombination during PCR. Nucleic Acids Res. 1990;18: 1687–1691. 2186361 10.1093/nar/18.7.1687 PMC330584

[25] Judo MS, Wedel AB, Wilson C. Stimulation and suppression of PCR-mediated recombination. Nucleic Acids Res. 1998;26: 1819–1825. 9512558 10.1093/nar/26.7.1819 PMC147471

[26] Pääbo S, Irwin DM, Wilson AC. DNA damage promotes jumping between templates during enzymatic amplification. J Biol Chem. 1990;265: 4718–4721. 2307682

[27] Odelberg SJ, Weiss RB, Hata A, White R. Template-switching during DNA synthesis by Thermus aquaticus DNA polymerase I. Nucleic Acids Res. 1995;23: 2049–2057. 7596836 10.1093/nar/23.11.2049 PMC306983

[28] Qiu X, Wu L, Huang H, McDonel PE, Palumbo AV, Tiedje JM, et al Evaluation of PCR-generated chimeras, mutations, and heteroduplexes with 16S rRNA gene-based cloning. Appl Environ Microbiol. 2001;67: 880–887. doi: 10.1128/AEM.67.2.880-887.2001 11157258 10.1128/AEM.67.2.880-887.2001 PMC92662

[29] Zylstra P, Rothenfluh HS, Weiller GF, Blanden RV, Steele EJ. PCR amplification of murine immunoglobulin germline V genes: strategies for minimization of recombination artefacts. Immunol Cell Biol. 1998;76: 395–405. doi: 10.1046/j.1440-1711.1998.00772.x 9797458 10.1046/j.1440-1711.1998.00772.x

[30] Acinas SG, Sarma-Rupavtarm R, Klepac-Ceraj V, Polz MF. PCR-induced sequence artifacts and bias: insights from comparison of two 16S rRNA clone libraries constructed from the same sample. Appl Environ Microbiol. 2005;71: 8966–8969. doi: 10.1128/AEM.71.12.8966-8969.2005 16332901 10.1128/AEM.71.12.8966-8969.2005 PMC1317340

[31] Lenz TL, Becker S. Simple approach to reduce PCR artefact formation leads to reliable genotyping of MHC and other highly polymorphic loci—Implications for evolutionary analysis. Gene. 2008;427: 117–123. doi: 10.1016/j.gene.2008.09.013 18848974 10.1016/j.gene.2008.09.013

[32] Wang GC, Wang Y. The frequency of chimeric molecules as a consequence of PCR co-amplification of 16S rRNA genes from different bacterial species. Microbiology. 1996;142 (Pt 5): 1107–1114. 8704952 10.1099/13500872-142-5-1107

[33] Thompson JR, Marcelino LA, Polz MF. Heteroduplexes in mixed-template amplifications: formation, consequence and elimination by “reconditioning PCR”. Nucleic Acids Res. 2002;30: 2083–2088. 11972349 10.1093/nar/30.9.2083 PMC113844

